# A retrospective cross-sectional study of risk factors for communicable disease diagnoses among refugees in mainland Greek camps, 2016–2017

**DOI:** 10.1038/s41598-024-65696-9

**Published:** 2024-07-02

**Authors:** Sarah Elizabeth Scales, Jee Won Park, Rebecca Nixon, Debarati Guha-Sapir, Jennifer A. Horney

**Affiliations:** 1 Epidemiology Program, University of Delaware, Newark, DE USA; 2 Department of Geography and Spatial Sciences, University of Delaware, Newark, DE USA; 3 Division of International Health, Johns Hopkins University Bloomberg School of Public Health, Baltimore, MD USA

**Keywords:** Refugee health, Complex emergencies, Communicable disease, Disease prevention, Risk factors

## Abstract

Communicable disease risk is high in refugee camps and reception centers. To better understand the risks for communicable disease diagnoses among refugees and asylum seekers, this study assesses individual- and camp-level risk factors among individuals utilizing Médecins du Monde clinics in four large refugee camps—Elliniko, Malakasa, Koutsochero, and Raidestos—on mainland Greece between July 2016 and May 2017. Descriptive statistics are reported for the demographic characteristics of the study population and for communicable disease burdens within the four camps—Elliniko, Malakasa, Raidestos, and Koutsochero. A hierarchical generalized linear model was used to assess risk factors for communicable disease diagnoses while accounting for individual-level clustering. This study shows marginal patterns in risk factors for communicable disease. Males had marginally higher risk of communicable disease diagnosis than females (OR = 1.12; 95% CI 0.97—1.29), and increased age was more protective against communicable disease for females (OR = 0.957; 95% CI 0.953—0.961) than for males (OR = 0.963; 95% CI 0.959—0.967). Communicable disease risk was significantly different between camps, with Elliniko (OR = 1.58; 95% CI 1.40–1.79) and Malakasa (OR = 1.43; 95% CI 1.25–1.63) having higher odds of communicable disease than Raidestos. The demographic and epidemiologic profiles of displaced populations differ across settings, and epidemiologic baselines for displaced populations are fundamental to evidence-informed provision of humanitarian aid. Further, while influences and risks for negative health outcomes in complex emergencies are broadly, the causal mechanisms that underpin these relationships are not as well understood. Both practitioners and researchers should engage with further research to elucidate the mechanisms through which these risks operate among displaced populations, including multilevel analyses.

## Introduction

Following the signing of the EU-Türkïye Deal and the informal closure of the Balkan Route that connected the Eastern Mediterranean transit routes to Western Europe, the mobility of refugees and asylum seekers in Greece was greatly constrained^1,2^. Despite the restrictions, crossings into Greece via the Aegean and Mediterranean Seas in March 2016 were roughly three times higher than they were at the same point in 2015, and unsanctioned crossings continued to significantly outpace sanctioned arrivals^3^. Greece, similar to many other European Union countries, was unable to scale-up their “hot-spots” to facilitate reception, registration, and eventual relocation of refugees and asylum seekers^4^. Together, these factors contributed to crowded and under supported camp and reception center accommodations for refugees.

Risks for outbreaks of communicable diseases are heightened in fragile contexts such as refugee camps and reception centers for newly arrived refugees and asylum seekers^5^. Altare et al.^6^ detailed the circumstances that contribute to infectious and communicable disease outbreaks in refugee camps, such as crowding, lack of water, sanitation, and hygiene (WASH) implementation and uptake, and constrained clinical access and provision of care^6^. The European Civil Protection and Humanitarian Aid Operations of the European Commission supported the mobilization of Médecins du Monde (MdM) teams to refugee camps and reception centers across mainland Greece to help mitigate health risks inherent to the escalating humanitarian crisis^7–9^. To better understand the health needs of refugees and asylum seekers in mainland camps and reception centers, the Hellenic Ministry of Health (MOH) set-up a syndromic surveillance system, with roughly 40 facilities reporting weekly number of cases for 14 syndromes. Throughout our study period, threshold warnings were issued for respiratory syndromes with fever, and non-bloody gastroenteritis, bloody diarrhea, rash with fever, and suspected scabies, when the observed proportional morbidity exceeded the expected proportional morbidity based on surveillance data from the previous 4-weeks^10^.

However, the majority of literature describing the aforementioned circumstances is from fragile settings across Africa and southeast Asia. The geography, weather, and climate, among other factors that also influence the natural history of communicable diseases in these settings, differ from tropical countries like those in Africa and southeast Asia to temperate countries in Europe^11^. Additionally, age and sex profiles differ across displaced populations and their respective situations. In the Eastern Mediterranean, refugees and asylum seekers are predominantly younger males, with nearly 70% of all male asylum seekers of non-European origin in 2015 under the age 35^12^. At the height of the European refugee crisis, unaccompanied minors—individuals under the age of 18 years—constituted upwards of 35% of all individuals entering Greece via Aegean and Mediterranean Sea crossings^13^. Accounting for the young age of refugees and asylum seekers is important for capturing the significant role age plays in communicable disease susceptibility and subsequent risk. Further, it is important to consider the age and sex simultaneously to better understand the influences of unmeasured sociocultural and religious practices as well as biological needs that influence health outcomes across both age and sex^14^.

Rojek et al.^5^, described both the challenges and opportunities for improvement in clinical data capture in Greek refugee camps as a means to better understand risks and facilitate outbreak preparedness in these settings. Although available clinical data does not always capture the totality of variables of interest, it is important to capitalize on the clinical data that are available from complex settings to contribute to the evidence base for data-informed decision-making for public health and resource provisions in refugee camps. Using data from MdM clinics from July 2016—May 2017, our study describes the burden of communicable diseases across four large refugee camps on the Greek mainland. Further, this study assesses the impact of individual and camp-level factors on communicable disease diagnosis among individuals with clinical consultations.

## Methods

### Study aim, description, and population

The aim of this study is to better understand the risks for communicable disease diagnoses among refugees and asylum seekers by assessing individual- and camp-level risk factors among individuals utilizing MdM clinics in four large refugee camps – Elliniko, Raidestos, Malakasa, and Koutsochero I and II – on mainland Greece between July 2016 and May 2017. Line-list data, including basic demographics and clinical notes, were collected at general, mental, sexual and reproductive, and dental health clinics. Within the four camps, there were 5190 individuals representing 19,044 consultations across clinics. The outcome of interest for this study was communicable disease diagnosis, defined as a binary variable for relative diagnoses of communicable versus non-communicable diagnoses.

### Data sources

Clinical data were collected by MdM clinicians and staff across camps from July 2016—May 2017. These data included camp, clinic, age, country of origin, diagnostic category, communicable/non-communicable, sex, diagnostic notes, and treatment notes. Because of the duration of time the camps were—and continue to be—in operation, a number of individuals had birthdays while in camps. The age at first consultation was used for demographic analysis, and age at specific consultation was used in regression models. Age was treated as a continuous variable. In accordance with Sendai Framework Monitor reporting standards for sex disaggregated data, sex is treated as a binary variable (male/female).

Camp level data were extracted from country, United Nations High Commissioner for Refugees (UNHCR), and International Organization for Migration (IOM) reports and briefings. WASH metrics, capacity, and distance to nearest health facility were provided for Elliniko and Koutsochero camps in UNHCR site profiles for 30 March 2016. For the purposes of this study, the three sites within Elliniko camp (Hockey Stadium; West/Olympic Arrivals; Baseball Stadium) were joined, with capacity and metrics reflective of the camps collectively. For Malakasa camp, general information is available from IOM for the 2016—2018 time period, after which Malakasa continued to evolve with changes in shelter unit types and service provisions. Data for camp capacity and provision of waste management services were retrieved from the report, *IOM in the Field: Improving the living conditions of migrants and refugees in mainland Greece 2016–2018*^15^. Latrine and shower units were obtained from July 2019 IOM site profiles (see limitations). Data were not available for WASH metrics from Raidestos camp, but narrative briefings from Central Macedonian camps indicated that WASH facilities and adherence were lacking. Therefore, we assumed that the number of latrines and showers per individual was below the Humanitarian Charter and Minimum Standards in Humanitarian Response (Sphere) threshold of 1 latrine for 20 people. There are a number of medical facilities within the greater Thermi area, so it was reasonable to assume that Raidestos camp was within a 5km distance of at least one health facility, in addition to having an MdM clinic on-site. Camp location and capacity were estimated and provided, respectively, through direct personal communication. Crowding was measured as the ratio of the total number of individual consultations and the provided capacity. WASH was measured as a binary variable (e.g., at least two WASH metrics (e.g., showers, latrines, and/or waste management)/ fewer than two metrics).

### Statistical analysis

Descriptive statistics were calculated using SAS Studio software (Cary, NC). Chi-square statistics were used for between and within camp comparisons of sex. A Kruskal–Wallis chi-square was used to compare median age across camps; median age with interquartile range is reported by camp. Plots were made in SAS Studio (Release 3.81) and Microsoft Excel (Redmond, Washington).

A hierarchical generalized linear model was fit using the GLIMMIX procedure using SAS Studio software^16^. The model was fit with a binomial distribution with non-communicable disease diagnoses as the reference level. Denominator degrees of freedom were specified using between/within which accounts for both between individual and within individual effects^17^. The random residual was specified with compound symmetry covariance structure; this structure supported model convergence and had the lowest − 2 residual log pseudo-likelihood when compared to other covariance structure specifications^18^. Random residuals were specified for repeated measures for individuals and the random intercept was specified for individual-level clustering. To investigate the role of camp-level explanatory factors, we specified an additional generalized linear model, accounting for clustering at both the individual (level-1) and camp (level-2) levels by partitioning variance components.

Bivariate analyses were conducted with both individual- and camp-level variables, and variables were included in the multi-level model if they had a bivariate *p*-value less than or equal to 0.2 and/or were considered important factors identified through literature review and subject-area expertise. Variables considered in bivariate analysis included age, sex, camp of consultation, and clinic of consultation. The final, multilevel model included age, sex, camp of consultation, and a product term for age and sex.

### Ethical approval

All methods were carried out in accordance with relevant guidelines and regulations. Secondary, deidentified data were used. This study was deemed to be exempt by the University of Delaware’s Institutional Review Board (2002123).

## Results

### Demographics

Demographics for individuals utilizing MdM clinical services in Elliniko, Raidestos, Koutsochero, and Malakasa are summarized in Table 1. Across camps, median age was not appreciably different. For camps in Attica, the median age was 20 years, and median age in Koutsochero and Raidestos was 16 and 17 years, respectively. There were roughly the same numbers of males and females with clinical consultations in Koutsochero, but there were appreciably more male consults for Elliniko, Malakasa, and Raidestos. A total of 13 states—Afghanistan, Algeria, Egypt, Iran, Iraq, Kuwait, Lebanon, Libya, Morocco, Pakistan, Occupied Palestine, Somalia, and Syria—were represented across the four camps. Elliniko and Malakasa hosted mostly Afghans while Koutsochero and Raidestos hosted predominantly Syrians. For the 5,190 individuals in the four camps, there were a total of 19,044 consultations (median = 2 visits; IQR = 3 visits; maximum number = 73). Nearly 34% of all consultations were for communicable diseases.

**Table 1:** Demographic characteristics for individuals (n = 5190) within and between camp.

Camp	Sex, n (%)			Age in years, median (25–75th percentiles)	
	Female	Male	*p*-value*		
Elliniko (n = 2002)	849 (42.4%)	1153 (57.6%)	< 0.0001	20 (8–30)	
Koutsochero (n = 879)	436 (49.6%)	443 (50.4%)	0.8134	16 (8–31)	
Malakasa (n = 1170)	516 (44.1%)	654 (55.9%)	< 0.0001	20 (8–31)	
Raidestos (n = 1139)	532 (46.7%)	607 (53.3%)	0.0263	17 (7–30)	
All camps (n = 5190)	2333 (44.95%)	2857 (55.05%)	0.0021	19 (8–30)	0.0622**

* *χ*2 *p*-value.

** Kruskal–Wallis *χ*2 *p*-value.

### Communicable disease consults

**Figure 1:**
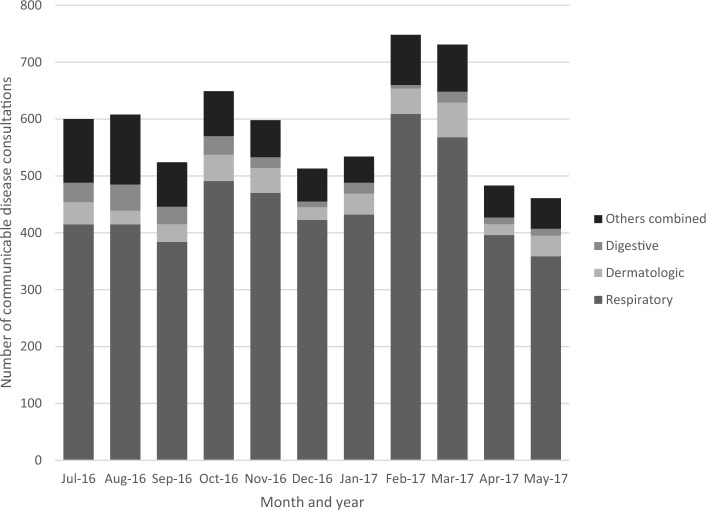
Respiratory consultations (76.94%) were the most common communicable disease consultations across camps, followed by dermatologic (6.26%) and digestive (3.74%). Others combined (13.06%) includes dental, general, ophthalmological, reproductive, and urology/nephrology communicable consultations. Frequencies are for total consultations (n = 19,044).

Diagnostic categories for communicable diseases for all camps and each camp specifically are summarized in Table 2. Monthly frequencies of communicable diseases are shown in Fig. 1. Nearly 80% of all communicable disease consultations were for respiratory infections (n = 4,962). Dermatological diagnoses (n = 404) were the next most common, accounting for 6.26% of all communicable disease consultations. General (n = 317) and digestive (n = 250) diagnoses accounted for just under 5% and 4% of diagnoses, respectively. Diagnostic category for communicable disease outcomes differed significantly across camps for all but dental consultations (Table 2). Communicable dental consultations constituted less than 2% of all communicable disease consults for each camp. Respiratory infections were the most frequent communicable diagnosis for all camps, constituting more than a third of all communicable diagnoses in each camp. While dermatological consults were the second most common for the entire study population, communicable digestive illness was the second most common consultation in Malakasa. In Raidestos, dermatological and digestive diseases were tied as the second most common communicable diagnoses.

**Table 2:** Frequency and percentage of diagnostic category for communicable disease consultations (n = 6449) for all camps and within individual camps.

Diagnostic Category	Elliniko		Koutsochero		Malakasa		Raidestos		All camps		
	n	%	n	%	n	%	n	%	n	%	Fisher's exact *p*-value
Dental	25	0.84%	8	1.54%	21	1.01%	4	0.46%	58	0.90%	0.19
Dermatology	219	7.35%	30	5.79%	94	4.51%	61	7.02%	404	6.26%	< 0.0001
Digestive	89	2.99%	10	1.93%	90	4.32%	61	7.02%	250	3.88%	< 0.0001
General	187	6.28%	2	0.39%	87	4.18%	41	4.72%	317	4.92%	< 0.0001
Ophthalmology	52	1.75%	6	1.16%	37	1.78%	45	5.18%	140	2.17%	< 0.0001
Reproductive Health	5	0.17%	26	5.02%	83	3.98%	41	4.72%	155	2.40%	< 0.0001
Respiratory	2357	79.12%	416	80.31%	1598	76.72%	591	68.01%	4962	76.94%	< 0.0001
Urology / Nephrology	45	1.51%	20	3.86%	73	3.50%	25	2.88%	163	2.53%	< 0.0001

*P*-values correspond to the Fisher’s exact test for frequencies between the 4 camps.

The results of the generalized linear model – accounting for clustering within camp – are shown in Table 3. The final model included age, sex, the product of age and sex, and camp of consultation. There was no significant difference in odds of communicable disease diagnosis between males and females (OR = 0.989; 95% CI 0.855–1.146). Every year increase in age from the population median of 19-years reduced the odds of communicable disease by 4.3% (OR = 0.957; 95% CI 0.953 – 0.961). The product term of age and sex showed little difference in communicable disease outcomes for males and females with increasing age (OR = 1.006; 95% CI 1.000 – 1.013).

**Table 3:** Odds ratios and 95% confidence intervals for the relationship between risk factors and clinical diagnoses using a clustered generalized linear model.

Variable name	OR	95% CI
Sex (Male vs. Female)	0.989	0.855–1.146
Age (Male vs. Female)	1.006	1.000–1.013
Age (continuous)	0.957	0.953–0.961
Camp (Elliniko vs. Raidestos)	1.580	1.398–1.786
Camp (Malakasa vs. Raidestos)	1.425	1.249–1.625
Camp (Koutsochero vs. Raidestos)	0.719	0.614–0.844

Odds ratios and 95% confidence intervals represent 1-year changes from the overall study population’s median age of 19 years.

The odds of communicable diseases were appreciably higher in some camps. Individuals with consultations at Elliniko (OR = 1.580; 95% CI 1.398 – 1.786) and Malakasa (OR = 1.425; 95% CI 1.249 – 1.625) were 58% and 42.5% more likely to have a communicable disease diagnosis, respectively, compared to individuals with consultations at Raidestos. Patients with consultations at Koutsochero were 28.1% less likely to have a communicable disease diagnosis compared to those at Raidestos (OR = 0.719; 95% CI 0.614 – 0.844). When using Elliniko as the reference camp, odds of communicable disease diagnosis were significantly lower for Koutsochero (OR = 0.455; 95% CI 0.396 – 0.524) and Raidestos (OR = 0.633; 95% CI 0.560 – 0.715) , while being similar for Malakasa (OR = 0.902; 95% CI 0.810 – 1.00).

To investigate the role of camp-level explanatory factors, we specified an additional generalized linear model, accounting for clustering at both the individual and camp level (Table 4). Crowding, measured as the ratio of consultations to a given camp’s reported capacity, was not predictive of individual-level communicable disease diagnosis (results not shown). In this model, males had slightly higher odds of communicable disease diagnoses than females (OR = 1.12; 95% CI 0.97—1.29). Every year increase from the median age of 19 years resulted in a 3.69% decrease in odds of communicable disease diagnoses for males when controlling for model covariates and accounting for individual- and camp-level clustering (OR = 0.963; 95% CI 0.959—0.967). For females, the odds of communicable disease diagnoses when controlling for model covariates and accounting for individual- and camp-level clustering were 4.31% lower for every year increase from the median age of 19 years (OR = 0.957; 95% CI 0.953—0.961). When accounting for age, sex, and the product term for age and sex, better WASH scores (e.g., meeting at least 2 out of 3 metrics for showers, latrines, and waste management) were marginally protective against communicable disease diagnoses compared to camps reporting implementation of only one or none of the Sphere standards (OR = 0.81; 95% CI 0.14—4.72). However, the limited variability in WASH measures, due in part to limited data availability, across camps limits the interpretation of these findings.

**Table 4:** Odds ratios and 95% confidence intervals for the relationship between individual- and camp-level factors and clinical diagnoses using a multi-level generalized linear model.

Variable name	OR	95% CI
Sex (Male vs. Female)	1.120	0.971–1.291
Age (Male)	0.963	0.959–0.967
Age (Female)	0.957	0.953–0.961
WASH (2–3 measures vs. 0–1 measures)	0.801	0.138–4.720

Odds ratios and 95% confidence intervals represent 1-year changes from the overall study population’s median age of 19 years.

## Discussion

### Context in Greece

In our study, with 76.94% of all communicable disease consultations and 26.06% of all-cause consultations being for respiratory infections. Similarly, week-to-week, Hellenic MOH weekly reports showed respiratory infections to have the highest proportional morbidity for refugees and asylum seekers. Respiratory infections are very common among displaced populations, with susceptibility driven by factors such as malnutrition, environmental exposures during transit, crowding and poor ventilation of housing accommodations, and underlying—often unregulated—chronic diseases^19–23^. Elliniko and Malakasa, which hosted predominantly Afghan refugees, had higher odds of communicable diseases, compared to mostly Syrian populations in Raidestos and Koutsochero. This could be partially explained by the comparatively higher level of epidemiologic transition experienced in pre-war Syria^24^ compared to Afghanistan^25,26^. Given the countries of origin for our populations and the physical environments of their temporary accommodations, the high proportional morbidity of respiratory tract infections is expected.

The proportional morbidity of conditions of interest in our study are consistent with those reported in other camp locations. For example, Kampouras et al., (2019), reported respiratory, dermatologic, urinary, and gastrointestinal infections as the most common communicable diseases among pediatric cases seen at in-camp primary clinics from October 2017 through March 2017^27^. In summer and autumn 2015 – prior to the period covered in this study—the majority of consultations at MSF clinics in Greece were for respiratory and skin infections, as well as trauma-related conditions^28^. Communicable diseases among our study population did not follow a seasonal pattern. This is consistent with findings from Louka et al., (2022)’s assessment of scabies infections among refugees and asylees in Greece, syndromic surveillance from the Hellenic MOH which did not demonstrate distinct seasonal patterns for any of the surveilled syndromes. This is reflected in the absence of seasonal patterns in monthly frequencies of communicable diseases within our study (Fig. 1).

### Age and sex

Year increases in age above the median age of 19 years reduced the odds of communicable disease diagnoses for our study populations. Across geographic locations and types of emergencies, children almost ubiquitously faced disproportionately negative impacts on health and wellbeing. This is particularly true among children from Afghanistan and Syria. In 2016, the under-5 mortality rate in Afghanistan was 67 deaths per 1000 live births, compared to 42 deaths per 1000 live births globally^29^. Acute lower respiratory infections were the leading cause of under-5 mortality in Afghanistan, excluding birth-related causes (i.e., birth trauma or asphyxia, prematurity)^30^. In addition to high numbers of direct deaths (i.e., deaths caused by explosions, remote violence, battles, violence against civilians)^31^, Syrian children affected by the civil war are disproportionately vulnerable to communicable diseases—including vaccine preventable diseases like hepatitis B, cholera, and measles—and malnutrition due to displacement^32–34^. For all communicable diseases, children.

Among our study population, males had slightly higher risks of communicable disease diagnosis compared to females. Increasing age was more protective against communicable disease diagnosis for women than for men. A large share of refugees and asylum seekers entering Greece at the time of our study were unaccompanied minor males^12^. Without cogent asylum and resettlement processes—especially for single males and male minors without family already in the European Union—these individuals remained in camps and reception centers beyond the intended timeline^35,36^. Although more males had all-cause consultations in Elliniko, Malakasa, and Raidestos camps, there is no evidence in our study to suggest that this is due to differential access to clinical care rather than a reflection of the sex distribution of camp populations. We did not have information on duration in camps, but this is an important factor to consider when explaining the age-sex differences in communicable disease risk among our study population.

### WASH

When assessing camp-level explanatory factors, our study did not find a clear relationship between camp-level WASH implementation in camps and individual-level risks of communicable disease diagnoses. This could, in part, be due to the limited number of camps in the study and resulting limited variability in WASH across camps. Further, while the metrics used in our study—provision of latrines, showers, and waste management—are vital for maintaining safe, clean, and dignified accommodations for displaced persons, these metrics do not cover the totality of WASH provisions that are needed. Additionally, when not assessing specific, targeted interventions, it is difficult to measure the impacts of WASH program in that WASH standards are not uniformly implemented across settings, and interventions and related recommendations vary and differ in uptake by community units and individuals^37,38^.

Given the underlying disease prevalences within our study population, we would not expect to see clear benefits from WASH implementation; the benefits of WASH are best measured and understood in the context of controlling pathogens that spread via water-borne and fecal–oral routes^38,39^. In our study, the proportional morbidity from communicable digestive illnesses was only 1.31% compared to nearly 80% for respiratory illnesses. However, there has not been extensive investigation into the impacts of WASH—particularly handwashing—on transmission of respiratory pathogens; this is especially true in lower-middle income countries^40,41^. Future studies should look at both individual-level WASH uptake and camp-level implementation of protocols to better understand the dynamics of WASH on respiratory infections in complex emergency settings.

### Limitations

There were several important limitations with assessing the role of variables of interest within a causal framework. A limited number of camps were used in the model to accommodate the need for adequate sample sizes and to address overdispersion of data. This challenge was particularly impactful for assessing camp-level variables, including WASH and capacity/crowding measures, in the individual- and camp-level clustering model. Further, the data used for WASH scores are from site and situation reports. There could be unintentional selection bias resulting from the use of only these camps rather than a more complete portfolio of Greek camps with MdM clinics.

For Raidestos and Malakasa, data were not available on WASH and municipal services for the study period. Situation briefs and subsequent reports were used to construct the aggregate WASH measure. Metrics for WASH and waste management can change frequently, and reports at one point might not be representative of the true circumstances on the ground over time. While there could be measurement bias associated with these choices, it is reasonable to assume that, with the dynamic nature of service provisions in refugee camps, the sources utilized provide a reasonable snapshot of experiences on the ground for at least some of the study period. The lack of total camp population estimates is not unique to our study^5,42^, but findings must be interpreted with the understanding that individuals in the analytic sample are only generalizable to those who are able to access and those who utilize in-camp clinical resources. Residual confounding could be present due to limited availability of additional individual- and camp-level variables.

## Conclusions

In our study, nearly 34% of all consultations across 4 large refugee camps and reception centers with MdM clinics on mainland Greece were for communicable diseases. Males had slightly higher risk of communicable disease diagnosis than women, and this difference was more pronounced when looking at age and sex simultaneously. This supports the United Nations Office for Disaster Risk Reduction’s emphasis on the need for sex and age disaggregated data reporting and analysis, and future work should further explore the mechanisms that underpin differences in communicable disease risk by age and sex. Despite measurement challenges, future research should continue to address multi-level risks—such as camp-level WASH implementation, crowding, and resource and support provisions—for communicable disease outbreaks in dynamic settings.

While this work contributes to the available literature on communicable diseases in refugee camps in mainland Greece at the height of the European refugee crisis, it also highlights areas that still need further attention, better data, and renewed interest. The preponderance of research investigating the health impacts and outcomes relating to displacement and living in refugee camp settings is centered on the African continent or in southeast Asia. With the increasingly complex global migration networks in the last decade, this research has expanded to include Western countries. However, there is still much work that needs to be done to understand the unique challenges both in terms of aid operationalization and risk matrices for communicable diseases among refugees and asylees in Europe.

## Data Availability

The datasets analyzed during the current study are not publicly available at the discretion of the original data holders, but data are available from the corresponding author upon reasonable request.

## Abbreviations

IOM: International organization for migration
MOH: Ministry of health
Sphere: Humanitarian charter and minimum standards in humanitarian response
UNHCR: United Nations high commissioner for refugees
WASH: Water, sanitation, and hygiene
